# *Molecular Cytogenetics*: the first impact factor (2.36)

**DOI:** 10.1186/1755-8166-6-28

**Published:** 2013-07-24

**Authors:** Thomas Liehr, Henry Heng, Yuri Yurov, Aurelia Meloni-Ehrig, Ivan Iourov

**Affiliations:** 1 Jena University Hospital, Friedrich Schiller University, Institute of Human Genetics, Jena, Germany; 2 Wayne State University School of Medicine, 540 E Canfield Street, Detroit, MI 48201, USA; 3 National Research Center of Mental Health, Russian Academy of Medical Sciences, Moscow, Russia; 4 Ameripath Florida, 8150 Chancellor Dr Ste 110, Orlando, FL 32809, USA

## 

We are pleased to announce that *Molecular Cytogenetics* has received its first official Impact Factor of 2.36 in June 2013.

We sincerely thank our distinguished international editorial board for their efforts on behalf of the journal, and our publisher, BioMed Central, for their in-house contribution to the speed and efficiency with which manuscripts are processed. Most important of all, of course, are our authors and reviewers [1], and to them we extend our particular thanks.

Since it was launched in 2008, *Molecular Cytogenetics* has considered 204 and published 179 articles, giving an acceptance rate of 86%. This exemplifies the journal’s inclusive editorial policy to publish manuscripts that are scientifically sound, and not just based on perceived impact. These articles cover a wide range of topics in molecular cytogenetics, including clinical and tumor cytogenetics, interphase architecture and karyotype evolution studies not only in human but also in other vertebrates. The journal is also open for molecular cytogenetic-based studies in any species, including invertebrates as well as plants. A rapid review process ensures that papers are published in a timely manner [1]. Our average time from submission to the first decision is 20 days, even under our stringent peer review process. As an open access journal, all papers are made immediately and freely available and articles are being accessed more than ever, with around 27,000 accesses per month. Table 1 shows the 10 most highly accessed articles published in *Molecular Cytogenetics* during the journal’s 5 years of publishing and Table 2 shows some additional highly cited articles. Authors can also access statistics about their article, such as how many times it has been accessed and discussed on social media websites. These statistics can be viewed by selecting ‘Article metrics’ in the right hand column of each article. In addition, all *Molecular Cytogenetics* articles are mobile-device optimized to facilitate easy reading on phones and tablets.

**Table 1:** **Highly assessed/cited papers published in *Molecular Cytogenetics *(information valid for the end of June 2013)**

**Reference**	**Title**	**Authors**	**Year/volume/number**	**Accesses**	**Citations**
[2]	On the origin of trisomy 21 Down syndrome	Maj A Hultén, Suketu D Patel, Maira Tankimanova, Magnus Westgren, Nikos Papadogiannakis, Anna Jonsson, Erik Iwarsson	*Molecular Cytogenetics* 2008, 1:21	15036	56
[3]	Human interphase chromosomes: a review of available molecular cytogenetic technologies	Svetlana G Vorsanova, Yuri B Yurov, Ivan Y Iourov	*Molecular Cytogenetics* 2010, 3:1	14502	35
[4]	FISH mapping of Philadelphia negative BCR/ABL1 positive CML	Anna Virgili, Diana Brazma, Alistair G Reid, Julie Howard-Reeves, Mikel Valgañón, Anastasios Chanalaris, Valeria AS De Melo, David Marin, Jane F Apperley, Colin Grace, Ellie P Nacheva	*Molecular Cytogenetics* 2008, 1:14	10908	19
[5]	Selection of single blastocysts for fresh transfer via standard morphology assessment alone and with array CGH for good prognosis IVF patients: results from a randomized pilot study	Zhihong Yang, Jiaen Liu, Gary S Collins, Shala A Salem, Xiaohong Liu, Sarah S Lyle, Alison C Peck, E Sills, Rifaat D Salem	*Molecular Cytogenetics* 2012, 5:24	9981	33
[6]	Mosaic 22q11.2 microdeletion syndrome: diagnosis and clinical manifestations of two cases	Ashutosh Halder, Manish Jain, Madhulika Kabra, Neerja Gupta	*Molecular Cytogenetics* 2008, 1:18	9728	17
[7]	Fluorescence in situ hybridization in combination with the comet assay and micronucleus test in genetic toxicology	Galina G Hovhannisyan	*Molecular Cytogenetics* 2010, 3:17	8963	16
[8]	The genome diversity and karyotype evolution of mammals	Alexander S Graphodatsky, Vladimir A Trifonov, Roscoe Stanyon	*Molecular Cytogenetics* 2011, 4:22	8794	11
[9]	Cytogenetic contribution to uniparental disomy (UPD)	Thomas Liehr	*Molecular Cytogenetics* 2010, 3:8	8348	28
[10]	The hierarchically organized splitting of chromosomal bands for all human chromosomes	Nadezda Kosyakova, Anja Weise, Kristin Mrasek, Uwe Claussen, Thomas Liehr, Heike Nelle	*Molecular Cytogenetics* 2009, 2:4	7881	5
[11]	Autistic disorder associated with a paternally derived unbalanced translocation leading to duplication of chromosome 15pter-q13.2: a case report	David J Wu, Nicholas J Wang, Jennette Driscoll, Naghmeh Dorrani, Dahai Liu, Marian Sigman, N Carolyn Schanen	*Molecular Cytogenetics* 2009, 2:27	7801	6

**Table 2:** Highly cited papers additional to those highly accessed

**Reference**	**Title**	**Authors**	**Year/volume/number**	**Citations**
[12]	Expanding the clinical phenotype of the 3q29 microdeletion syndrome and characterization of the reciprocal microduplication	Blake C Ballif, Aaron Theisen, Justine Coppinger, Gordon C Gowans, Joseph H Hersh, Suneeta Madan-Khetarpal, Karen R Schmidt, Raymond Tervo, Luis F Escobar, Christopher A Friedrich, Marie McDonald, Lindsey Campbell, Jeffrey E Ming, Elaine H Zackai, Bassem A Bejjani, Lisa G Shaffer	*Molecular Cytogenetics 2008, 1:8*	117
[13]	Chromosomal mosaicism goes global	Ivan Y Iourov, Svetlana G Vorsanova, Yuri B Yurov	*Molecular Cytogenetics* 2008, 1:26	74
[14]	Microdeletion of 6q16. 1 encompassing EPHA7 in a child with mild neurological abnormalities and dysmorphic features: case report	Traylor RN, Fan Z, Hudson B, Rosenfeld JA, Shaffer LG, Torchia BS, Ballif BC	*Molecular Cytogenetics* 2009, 2:17	41
[15]	Comparative analysis of copy number detection by whole-genome BAC and oligonucleotide array CGH	Neill NJ, Torchia BS, Bejjani BA, Shaffer LG, Ballif BC	*Molecular Cytogenetics* 2010, 3:11	40
[16]	Cryptic genomic imbalances in patients with de novo or familial apparently balanced translocations and abnormal phenotype	Sismani C, Kitsiou-Tzeli S, Ioannides M, Christodoulou C, Anastasiadou V, Stylianidou G, Papadopoulou E, Kanavakis E, Kosmaidou-Aravidou Z, Patsalis PC	*Molecular Cytogenetics* 2008, 1:15	39
[17]	Chromosome distribution in human sperm - a 3D multicolor banding-study	Manvelyan M, Hunstig F, Bhatt S, Mrasek K, Pellestor F, Weise A, Simonyan I, Aroutiounian R, Liehr T	*Molecular Cytogenetics* 2008, 1:25	34
[18]	GIN'n'CIN hypothesis of brain aging: deciphering the role of somatic genetic instabilities and neural aneuploidy during ontogeny	Yurov YB, Vorsanova SG, Iourov IY	*Molecular Cytogenetics* 2009, 2:23	29

Recently an obituary of a key person in our field, Prof. Dr. Lore Zech, inventor of banding cytogenetics was published and highly recognized in the community [19]. To reflect the needs of our community, in addition to publishing articles recognizing key contributions from the field of molecular cytogenetics, we are very pleased to launch our new journal blog to promote interaction among researchers. In this blog, we will engage in specific discussions with regards to new directions in the field and newly emerging technologies, as well as feedback from recent meetings/articles and more. The blog can be accessed at [20], and also through the journal homepage.

The field of molecular cytogenetics is now entering an exciting time. Following various large-scale sequencing projects that have revealed a high level of genetic heterogeneity, it is clear that single-cell resolution and cell population dynamics are essential to understanding the mechanisms of many common and complex diseases. Thus molecular cytogenetics has become increasingly important as it provides information on both individual cells and cell populations. We hope our journal *Molecular Cytogenetics* will promote the popularity of the molecular cytogenetic approach during the post-genomic era.

With the help of its readers and authors we look forward to the continuing growth of *Molecular Cytogenetics*.

## References

[B1] Liehr T Heng H Yurov YB Reviewer acknowledgement 2013 Mol Cytogenet 2013 6 9 10.1186/1755-8166-6-923587462

[B2] Hultén MA Patel SD Tankimanova M Westgren M Papadogiannakis N Jonsson AM Iwarsson E On the origin of trisomy 21 down syndrome Mol Cytogenet 2008 1 21 10.1186/1755-8166-1-21 18801168 PMC2564957

[B3] Vorsanova SG Yurov YB Iourov IY Human interphase chromosomes: a review of available molecular cytogenetic technologies Mol Cytogenet 2010 3 1 10.1186/1755-8166-3-1 20180947 PMC2830939

[B4] Virgili A Brazma D Reid AG Howard-Reeves J Valgañón M Chanalaris A De Melo VA Marin D Apperley JF Grace C Nacheva EP FISH mapping of Philadelphia negative BCR/ABL1 positive CML Mol Cytogenet 2008 1 14 10.1186/1755-8166-1-14 18638369 PMC2500019

[B5] Yang Z Liu J Collins GS Salem SA Liu X Lyle SS Peck AC Sills ES Salem RD Selection of single blastocysts for fresh transfer via standard morphology assessment alone and with array CGH for good prognosis IVF patients: results from a randomized pilot study Mol Cytogenet 2012 5 24 10.1186/1755-8166-5-24 22551456 PMC3403960

[B6] Halder A Jain M Kabra M Gupta N Mosaic 22q11.2 Microdeletion syndrome: diagnosis and clinical manifestations of two cases Mol Cytogenet 2008 1 18 10.1186/1755-8166-1-18 18691436 PMC2527005

[B7] Hovhannisyan GG Fluorescence in situ hybridization in combination with the comet assay and micronucleus test in genetic toxicology Mol Cytogenet 2010 3 17 10.1186/1755-8166-3-17 20840797 PMC2949878

[B8] Graphodatsky AS Trifonov VA Stanyon R The genome diversity and karyotype evolution of mammals Mol Cytogenet 2011 4 22 10.1186/1755-8166-4-22 21992653 PMC3204295

[B9] Liehr T Cytogenetic contribution to uniparental disomy (UPD) Mol Cytogenet 2010 3 8 10.1186/1755-8166-3-8 20350319 PMC2853554

[B10] Kosyakova N Weise A Mrasek K Claussen U Liehr T Nelle H The hierarchically organized splitting of chromosomal bands for all human chromosomes Mol Cytogenet 2009 2 4 10.1186/1755-8166-2-4 19171032 PMC2636822

[B11] Wu DJ Wang NJ Driscoll J Dorrani N Liu D Sigman M Schanen NC Autistic disorder associated with a paternally derived unbalanced translocation leading to duplication of chromosome 15pter-q13.2: a case report Mol Cytogenet 2009 2 27 10.1186/1755-8166-2-27 20021661 PMC2803171

[B12] Ballif BC Theisen A Coppinger J Gowans GC Hersh JH Madan-Khetarpal S Schmidt KR Tervo R Escobar LF Friedrich CA McDonald M Campbell L Ming JE Zackai EH Bejjani BA Shaffer LG Expanding the clinical phenotype of the 3q29 microdeletion syndrome and characterization of the reciprocal microduplication Mol Cytogenet 2008 1 8 10.1186/1755-8166-1-8 18471269 PMC2408925

[B13] Iourov IY Vorsanova SG Yurov YB Chromosomal mosaicism goes global Mol Cytogenet 2008 1 26 10.1186/1755-8166-1-26 19032785 PMC2612668

[B14] Traylor RN Fan Z Hudson B Rosenfeld JA Shaffer LG Torchia BS Ballif BC Microdeletion of 6q16.1 encompassing EPHA7 in a child with mild neurological abnormalities and dysmorphic features: case report Mol Cytogenet 2009 2 17 10.1186/1755-8166-2-17 19664229 PMC2731778

[B15] Neill NJ Torchia BS Bejjani BA Shaffer LG Ballif BC Comparative analysis of copy number detection by whole-genome BAC and oligonucleotide array CGH Mol Cytogenet 2010 3 11 10.1186/1755-8166-3-11 20587050 PMC2909945

[B16] Sismani C Kitsiou-Tzeli S Ioannides M Christodoulou C Anastasiadou V Stylianidou G Papadopoulou E Kanavakis E Kosmaidou-Aravidou Z Patsalis PC Cryptic genomic imbalances in patients with de novo or familial apparently balanced translocations and abnormal phenotype Mol Cytogenet 2008 1 15 10.1186/1755-8166-1-15 18644119 PMC2516517

[B17] Manvelyan M Hunstig F Bhatt S Mrasek K Pellestor F Weise A Simonyan I Aroutiounian R Liehr T Chromosome distribution in human sperm - a 3D multicolor banding-study Mol Cytogenet 2008 1 25 10.1186/1755-8166-1-25 19014589 PMC2613144

[B18] Yurov YB Vorsanova SG Iourov IY GIN'n'CIN hypothesis of brain aging: deciphering the role of somatic genetic instabilities and neural aneuploidy during ontogeny Mol Cytogenet 2009 2 23 10.1186/1755-8166-2-23 19939257 PMC2787505

[B19] Schlegelberger B In memoriam: Prof. Dr. rer. nat. Dr. med. h.c. Lore Zech; 24.9.1923 - 13.3.2013: honorary member of the European society of human genetics, honorary member of the German society of human genetics, doctor laureate, the University of Kiel, Germany Mol Cytogenet 2013 6 20 10.1186/1755-8166-6-20 23693023 PMC3660208

[B20] Molecular cytogenetics bloghttp://blogs.biomedcentral.com/molcyt

